# Whole-Exome Sequencing on Circulating Tumor Cells Explores Platinum-Drug Resistance Mutations in Advanced Non-small Cell Lung Cancer

**DOI:** 10.3389/fgene.2021.722078

**Published:** 2021-09-20

**Authors:** Yuanyuan Chang, Yin Wang, Boyi Li, Xingzhong Lu, Ruiru Wang, Hui Li, Bo Yan, Aiqin Gu, Weimin Wang, Aimi Huang, Shuangxiu Wu, Rong Li

**Affiliations:** ^1^Department of Pulmonary Medicine, Shanghai Chest Hospital, Shanghai Jiao Tong University, Shanghai, China; ^2^Clinical Research Center, Shanghai Chest Hospital, Shanghai Jiao Tong University, Shanghai, China; ^3^Berry Oncology Corporation, Beijing, China

**Keywords:** circulating tumor cells, drug resistance, non-small cell lung cancer, platinum-based chemotherapy, single cell–level WES

## Abstract

Circulating tumor cells (CTCs) have important applications in clinical practice on early tumor diagnosis, prognostic prediction, and treatment evaluation. Platinum-based chemotherapy is a fundamental treatment for non-small cell lung cancer (NSCLC) patients who are not suitable for targeted drug therapies. However, most patients progressed after a period of treatment. Therefore, revealing the genetic information contributing to drug resistance and tumor metastasis in CTCs is valuable for treatment adjustment. In this study, we enrolled nine NSCLC patients with platinum-based chemotherapy resistance. For each patient, 10 CTCs were isolated when progression occurred to perform single cell–level whole-exome sequencing (WES). Meanwhile the patients’ paired primary-diagnosed formalin-fixed and paraffin-embedded samples and progressive biopsy specimens were also selected to perform WES. Comparisons of distinct mutation profiles between primary and progressive specimens as well as CTCs reflected different evolutionary mechanisms between CTC and lymph node metastasis, embodied in a higher proportion of mutations in CTCs shared with paired progressive lung tumor and hydrothorax specimens (4.4–33.3%) than with progressive lymphatic node samples (0.6–11.8%). Functional annotation showed that CTCs not only harbored cancer-driver gene mutations, including frequent mutations of *EGFR* and *TP53* shared with primary and/or progressive tumors, but also particularly harbored cell cycle–regulated or stem cell–related gene mutations, including *SHKBP1*, *NUMA1*, *ZNF143*, *MUC16*, *ORC1*, *PON1*, *PELP1*, etc., most of which derived from primary tumor samples and played crucial roles in chemo-drug resistance and metastasis for NSCLCs. Thus, detection of genetic information in CTCs is a feasible strategy for studying drug resistance and discovering new drug targets when progressive tumor specimens were unavailable.

## Introduction

Lung cancer is the most common malignancy with the highest incidence and mortality both in China and worldwide (Siegel et al., 2021). Non-small cell lung cancer (NSCLC) comprises about 85% of all lung cancers (LCs), and 50% of NSCLC patients relapse within 5 years after surgery (Taylor et al., 2012; Uramoto and Tanaka, 2014). With the development of detection techniques and targeted therapies, the diagnosis and treatment of NSCLC have made great progress and increased patients’ 5-year survival rate in recent years (Carlisle et al., 2020; Siegel et al., 2021). Postoperative adjuvant platinum-based chemotherapy is still the standard regimen for NSCLC patients for whom targeted therapy or progressive after targeted therapy is not applicable. However, a great proportion of NSCLC patients still progress to late-stage disease because of development of drug resistance after treatment. Therefore, it is important to reveal the genetic features of such drug resistance and adjust subsequent treatment to meet precision medicine targets.

Circulating tumor cells (CTCs) are cancer cells detected in the blood of cancer patients. They are assumed to disseminate from the primary tumor lesions and carry the primary lesion’s genetic information to relapse tissues through blood circulation during tumor development (Alix-Panabières and Pantel, 2013). CTC’s tumorigenic potential is validated in immune-compromised mice by using CTCs from breast cancer, NSCLC, small cell lung cancer (SCLC), and melanoma (Baccelli et al., 2013; Hodgkinson et al., 2014; Girotti et al., 2016; Morrow et al., 2016). It is increasingly accepted to use CTCs in monitoring of early tumor prognosis, recurrence assessment, and therapeutic drug screening in many cancers (Lin et al., 2018). For example, CTCs can be cultured *in vitro* for drug screening (Yu et al., 2014; Carter et al., 2017) or explanted in immune-compromised mice for different therapeutic interventions in SCLC (Hodgkinson et al., 2014; Drapkin et al., 2018) and NSCLC (Morrow et al., 2016; Xu et al., 2018a). Therefore, genetic information in CTCs from drug-resistance patients can help to dissect the mutational evolutionary process driving the transition from a primary tumor to a progressive or metastatic tumor during drug treatment and help us to speculate on the drug-resistance mechanisms underlying tumor progression and explore new drug targets.

In this study, we enrolled a total of nine patients of stage II–IV NSCLC who were all treated with platinum-based chemotherapy independently since diagnosis or after targeted drug treatment, and all progressed within 3 years after chemotherapy (Table 1 and Supplementary Table 1). For each patient at the tumor progressive phase, 10 ml of peripheral blood samples and paired lymph node or lung tumor tissue biopsy or 20 ml of hydrothorax were collected in addition to the paired primary formalin-fixed and paraffin-embedded (FFPE) lung tumor or hydrothorax samples when diagnosed. Then CTCs were isolated from the blood samples using a ClearCell FX1 platform. Whole-exome sequencing (WES) was performed on CTCs and paired primary tumor and progressive samples for each patient to compare the difference of somatic mutation profiles to reveal the genetic information involved in platinum-based chemotherapy and progression of NSCLC.

**TABLE 1:** Detailed clinical information for each patient.

**Patient ID**	**Gender**	**Age (y)**	**Smoking status**	**Pathology type**	**Tumor stage** ^¶^	**Chemotherapy drugs**	**Chemo-therapy cycle**
P1	Female	59	Never-smoker	ADC	II/III	Pemetrexed + Carboplatin	4
P2	Male	57	Current smoker	ADC	II/III	Pemetrexed + Carboplatin	8
P3	Male	63	Current smoker	SCC	IV/IV	Gemcitabine + nedaplatin	4
P4	Female	64	Never-smoker	ADC	IV/IV	Pemetrexed + carboplatin	8
P5	Female	66	Never-smoker	ADC	II/IV	Pemetrexed + carboplatin	5
P6	Male	56	Current smoker	ADC	IV/IV	Pemetrexed + carboplatin	4
P7	Female	53	Never-smoker	ADC	III/III	Pemetrexed + carboplatin	4
P8	Male	52	Current smoker	ADC	III/III	Pemetrexed + carboplatin	4
P9	Male	58	Current smoker	SCC	III/IV	Gemcitabine + nedaplatin	4

*^¶^ Information before the forward slash (/) refers to the primary-tumor stage, and that after the forward slash (/) refers to the progressive-sample stage. ADC, adenocarcinoma carcinoma; SCC, squamous cell carcinoma; y, years.*

## Materials and Methods

### Patients and Sampling

Nine relapsed NSCLC patients (Stage II–IV) after treating with platinum-based chemotherapy were enrolled in this study. Their peripheral blood samples, paired tumor biopsies, or hydrothorax samples at disease progressive stage as well as FFPE samples of primary tumor tissues were collected at the Shanghai Chest Hospital of Shanghai Jiao Tong University. Diagnoses of LC were histopathologically confirmed for all patients. Clinical and pathological information are summarized in Supplementary Table 1. Informed consent, for sample, acquisition and research purposes in this study were obtained from all patients, and the study was approved by the Medical Ethics Committee of Shanghai Chest Hospital of Shanghai Jiao Tong University to conduct in accordance with the Declaration of Helsinki principles.

### Isolation of Circulating Tumor Cells From Blood Samples

**FIGURE 1:**
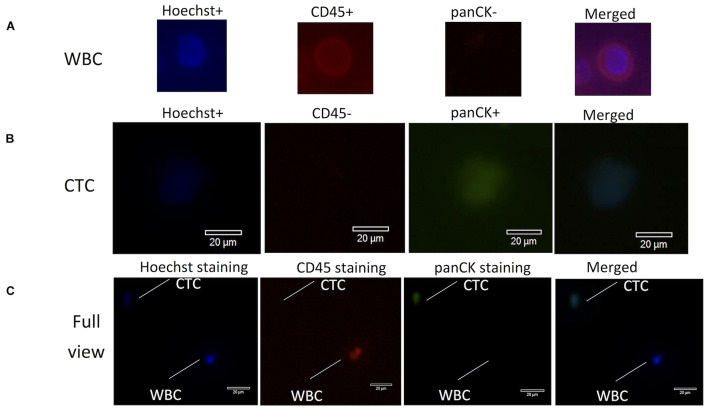
Fluorescence images for identification of CTCs and WBCs stained with antibodies from lung cancer patients. **(A)** WBCs were stained with Hoechst (blue), CD45+ (red), panCK– (dark), and merged images of three-antibody staining. **(B)** CTCs were stained with Hoechst (blue), CD45– (dark), panCK+ (green), and merged images of three-antibody staining. **(C)** Optical images (60× magnification) of isolated CTCs and WBCs under the microscope. Scale bar is 20 mm. CTC morphology is characterized by green fluorescence staining by panCK and nuclear blue staining by Hoechst but negative staining by CD45. CTCs, circulating tumor cells; WBCs, white blood cells.

A total sample of 10 ml of blood from each cancer patient was collected in a Streck vacutainer tube and processed within 24 h after collection. CTCs were first enriched *via* mass-dependent microfluidics and then manually picked through immunofluorescent staining of both positive staining for pan-cytokeratins (panCK+) and negative staining for CD45 (CD45−) on the basis of an intact Hoechst-stained nucleus (blue fluorescence) as in the method previously reported (Hou et al., 2013; Xu et al., 2018a; Abouleila et al., 2019; Chemi et al., 2019). Anti-CD45 (AB40763, Abcam, United States), anti-panCK (AB7753, Abcam, United States), and Hoechst 33342 were used for imaging of CTCs and white blood cells (WBCs) (Figure 1), which were counted under a fluorescence microscope (Olympus IX73), manually picked with a glass capillary into an ice-precooling low-adsorption PCR tube, and stored at −80°C for further experiments. To prevent sequencing failures, biological triplicates to quintuplicates of 10 CTCs were collected from each patient. Biological triplicates of 10 WBCs were collected from the blood sample of P8 and used as the reference control for calling mutations in 10-CTC WES data sets after whole genome amplification (WGA).

### Genomic DNA Isolation and Purification From Bulk Tumor Cell Samples

Genomic DNA (gDNA) was extracted from the FFPE samples using the GeneRead DNA FFPE Kit (Qiagen, United States), from the fresh tumor tissue samples using AllPrep DNA/RNA Mini (Qiagen, United States), from bulk WBC samples using the DNA Blood Midi/Mini kit (Qiagen, United States), or large DNA fragments (>500 bp) from the hydrothorax samples using the MagMAX^TM^ Cell-Free DNA Isolation Kit (Life Technology, United States) according to the manufacturer’s instructions, respectively. The quality of purified DNA was assayed by gel electrophoresis and quantified by a Qubit^®^ 4.0 Fluorometer (Life Technologies, United States).

### Oligo-Cell Whole Genome Amplification

After PBS buffer was removed by centrifugation, 10 CTCs or WBCs were directly lysed in the PCR tube and WGA was performed using the kits of both Preimplantation Genetic Screening of Chromosomal Copy Number Variations (Berry Genomics, China) and/or MALBAC Single Cell DNA Quick-Amp Kit (Yikongenomics, China) according to the manufacturer’s protocols. The WGA gDNA was further underwent WES according to the following method.

### Library Construction and Whole-Exome Sequencing

The purified gDNA or WGA gDNA was first fragmented into DNA pieces around 300 bp using the enzymatic method (5 × WGS Fragmentation Mix, Qiagen, United States). The WES library was prepared and constructed using the 96 rxn xGen Exome Research Panel v1.0 (Integrated DNA Technologies, United States), and sequencing was applied on a NovaSeq 6000 platform (Illumina, San Diego, CA, United States) with a 150PE mode according to the manufacturer’s protocol (Jia et al., 2020).

### Bioinformatics Analysis of Standard Whole-Exome Sequencing Data

For the tumor tissue or hydrothorax samples, single nucleotide variations (SNVs) and small insertions and deletions (Indels) were called and annotated according to previous reports (Jia et al., 2020). Briefly, the non-synonymous SNVs/Indels with VAF > 3% or with VAF > 1% in cancer hot spots as well as with VAF ratio of variant/reference >5 and *p* < 0.01 (Fisher’s exact test) for the variant compared between tumor samples and WBC samples were kept for further analysis (Supplementary Table 3). Tumor mutation burden (TMB) was defined as the total number of non-synonymous SNVs/Indels per megabase of coding region of a tumor genome in WES.

### Bioinformatics Analysis of Whole-Exome Sequencing Data of Circulating Tumor Cells

To obtain high-confidence SNVs/Indels from WES of CTCs, the following filters were applied in addition to the above analysis, and the final results are listed in Supplementary Table 3:

(1) Based on annotations using ANNOVAR (Wang et al., 2010), the variants that presented in either the 1000 genomes or the ExAC 03 database were removed.
(2) Mutations that referred to blacklisted genomic regions obtained from the UCSC Genome Table Browser and ENCODE Data Analysis Consortium were removed (Letouzé et al., 2017).
(3) Any variants called in any type of the WBC controls were also excluded.
(4) Variants in CTCs that also presented in either the tumor tissue or malignant pleural effusion samples were kept for further analysis.
(5) After the above filtration, for each patient, if a variant presented in tumor tissues or malignant pleural effusion samples but was not covered in the CTC WES data sets, its read number and sequencing depth were checked in the bam file of CTCs. If the variant reads ≥4 in CTCs and <0.01 (Fisher’s exact test) for the variants compared between CTCs and tumor samples, the variant was considered as a true mutation in CTCs and kept for further analysis. If *p* > 0.05, the missing variant in CTCs might be caused by low sequencing coverage in CTC WES, and it was removed from further analysis.

### Chemo-Resistance and Stem Cell–Related Mutation Screening

For the genes and variants related to cancer chemotherapy treatment, we downloaded a file, Variant, Gene, and Drug Relationship Data.zip, from PharmGKB annotations^1^ as the reference data set and blasted against the variants calling in all the samples to obtain the chemo-resistance genes and mutations list. For the genes and variants related to the function of stem cells, the mutations were screened by the keywords of “stem cell” in the annotation results and then we manually retrieved their influence on cancer stem cells in the published literatures. In addition, all the mutations of each sample were also blasted against the gene lists of 10 canonical oncogenic signaling pathways and 10 DNA damage repair pathways according to the published The Cancer Genome Atlas (TCGA) methods (Knijnenburg et al., 2018; Sanchez-Vega et al., 2018) because these genes are usually cancer-driver genes and contribute to tumor metastasis and drug resistance. These screening results are also listed in Supplementary Table 3.

### Statistical Analysis and Data Visualization

Statistical analysis used in mutation calling in the CTCs and tumor tissues was described as in the above methods. The Venn and heat map of data visualization were conducted by R/Bioconductor software packages.^2^

## Results

### Comparison of Whole-Exome Sequencing Quality of Oligo Circulating Tumor Cells, White Blood Cells, and Tumor Specimens

To sequence more CTCs each time as well as to consider the threshold of calling somatic mutations, we pooled 10 CTCs or WBCs together in one tube each time to construct a sequencing library and performed the single cell–level WES as described in the method section. The sequencing data first had filtered out the low-quality reads. More than 83% effective reads were mapped to the reference human genome sequence (hg19/GRCh37) for each sample; the mean coverage of whole exome target regions was 94.8% for CTCs and 87.9% for WBCs as well as 20× depth coverage that accounted for about 87.4 and 61.0% of the whole exome regions for CTCs and WBCs, respectively (Supplementary Table 2), similar to the previous report (Chemi et al., 2019). For the WES of primary and progressive tumor or malignant pleural effusion specimens of each patient, at least 93% effective reads were mapped to the reference human genome sequence, and 20× depth reads covered more than 98.3% of the whole exome regions (Supplementary Table 2).

Single nucleotide variations/Indels called in CTCs were filtered by those detected in 10 WBCs and bulk WBCs; only SNVs/Indels that were also detected in primary or progressive specimens or hydrothorax samples were involved for further bioinformatics analysis (Supplementary Table 3) as described in the method section.

### Various Heterogeneity of Somatic Mutations During Tumor Evolution After Platinum-Based Chemotherapy

**FIGURE 2:**
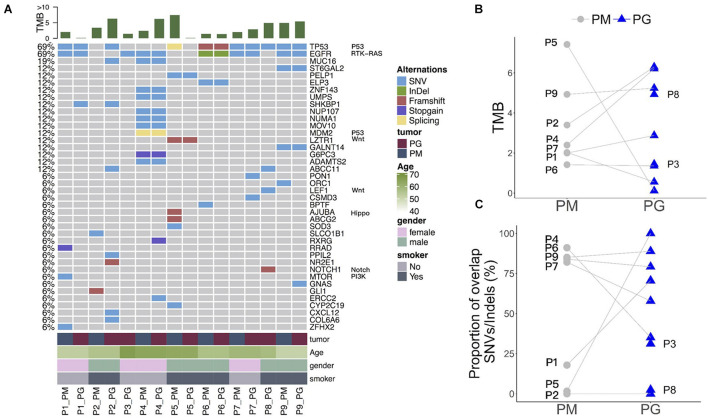
Somatic mutation spectrum and changes of mutation abundance before and after platinum-based chemotherapy treatment of each patient. **(A)** Somatic mutation spectrum in PM and PG. TMB, gene names and related pathways, and clinical information of each patient are on the top, right, and left of the graph. **(B)** Changes of TMB in PM and PG. **(C)** Shared gene mutation proportion in PM and PG. PM, primary specimens; PG, progressive specimens; TMB, tumor mutations burden.

Whole-exome sequencing data show a distinct somatic mutation spectrum of primary and progressive specimens with platinum-drug resistance features (top 40 genes in Figure 2A and Supplementary Table 3). The progressive mutational gene functions were significantly enriched to cancer-driver, cell adhesion, and metal ion/calmodulin/ATP binding genes in comparison with those in primary tumors: calcium signaling and cell cycle pathways, protein kinase, transcription factor activity, and calmodulin/ATP/chromatin bindings (Supplementary Table 4). Except for patients P3 and P8, who had no paired primary FFPE sample, more than half of patients’ (*n* = 5/7) primary and progressive tumors harbored dozens of overlap mutations, variously accounting for from 16.7 up to 76.9% of total mutations in each patient, indicating various somatic mutation heterogeneity during tumor evolution after platinum-based chemotherapy in different patients, which could also be reflected by TMB changes. Five of seven patients (71.4%) harbored increased TMB (Figure 2B) and three (42.9%) had less proportion of shared gene mutations (Figure 2C) in the progressive specimens than in the primary ones, indicating that new mutations occurred in the progressive tumors when drug resistance occurred in these patients.

The overlapped mutations between primary and progressive tumor tissues included LC–driver genes (Sanchez-Vega et al., 2018), such as recurrent mutations of *EGFR* and *TP53* among most patients as well as sporadic mutations of essential genes *FAT1*, *NOTCH2*, *NTHL1*, and *POLH* (of P4), *RB1*, *MSH6*, *ATR*, *ATRIP*, and *APTX* (of P6), *SMAD4* (of P7), *CDKN1A*, *RBL1*, *FZD10*, *PER1, PPP4R4*, and *FANCM* (of P9) as well as multidrug resistance genes of *MDM2*, *NUP107*, and *UMPS* (of P4) (Figure 2A and Supplementary Table 3), showing the diversity of somatic mutations during tumor metastasis and platinum-drug resistance among different patients.

### Circulating Tumor Cells Harboring Mutations Exclusively From Those in Progressive Lymph Nodes

**FIGURE 3:**
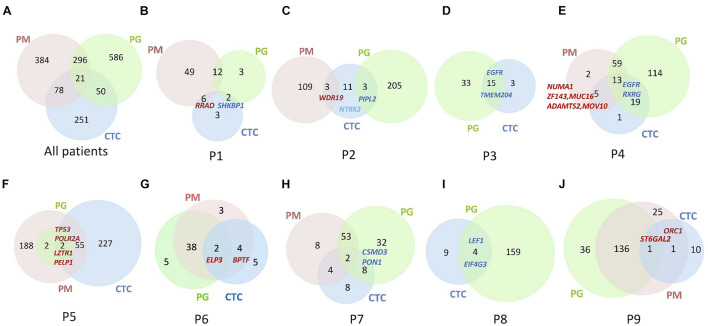
Venn diagrams to show numbers of SNVs/Indels identified in the lung PM, PG, and CTC specimens. **(A)** Summary of all nine patients. **(B–J)** Each patient from Patient 1 to 9. Numbers in Venn diagrams referred to gene numbers. Some essential mutation genes identified are indicated at the corresponding samples. Gene names in bright red denote the mutations shared among PM, PG, and CTC specimens; gene names in dark red denote shared between primary specimens and CTCs; gene names in blue denote shared between progressive specimens and CTC.

The sequencing results show 10 CTCs carried dozens of SNVs/Indels on an overall level, and the mutation abundance in CTCs varied in different patients (Figure 3). Only one patient’s CTC carried more and exclusive SNVs/Indels in comparison with the paired primary and progressive tumor specimens (*n* = 1/9, P5; Figure 3F). Other patients harbored a small number of SNVs/Indels simultaneously identified in CTCs and primary and progressive tumor samples, in total accounting for 2.69% of primary and 2.20% of progressive tumor mutations, respectively, indicating a high level of tumor heterogeneity among CTCs and primary and progressive specimens. Notably, the CTCs of three patients (P3, P4, and P6) whose primary and progressive tumor specimens were lung tumor tissue or hydrothorax harbored more mutations shared with progressive samples (33.3% for lung tumor tissue and 4.4–15.6% for hydrothorax) than with progressive lymph nodes (0.6–11.8%). Particularly for P4, the shared mutations included several important tumor metastasis–related and drug-resistance genes, such as *ZNF143*, *MUC16*, *NUMA1*, *ADAMTS2*, and *MOV10* (Figure 3E and Supplementary Table 3).

Comparing mutations of CTCs with those of primary or progressive samples, except for P2 whose CTCs contained the same numbers of shared mutations between primary and progressive specimens, included a cancer-suspicious gene *WDR19* in primary tumors and a tumor suppressor gene *PPIL2* in progressive lymph nodes. Four patients’ CTCs (*n* = 4/7, 57.1%) harbored more mutation overlap with the primary lung tumors, including a nonsense mutation of a tumor-suppressor gene *RRAD* in P1, cancer-driver genes of *POLR2A* and *TP53* in P5, chromatin-remodeling oncogenes *BPTF* and *ELP3* in P6, a stem cell–related gene *ORC1*, and a drug-resistant gene *ST6GAL2* in P9 (Figure 3 and Supplementary Table 3). Two patients’ CTCs, including one progressive specimen that was a hydrothorax biopsy (*n* = 1/2, 50.0%) and one progressive specimen that was a lymph node biopsy (*n* = 1/6, 16.7%), harbored mutation profiles more like those of progressive specimens, including chemotherapy-related gene *RXRG* and *EGFR* (with a new mutation site of p.L718Q) in P4 as well as drug-resistance genes *ARHGAP26*, *CSMD3*, and a stem cell–related gene *PON1* in P7. Our findings are consistent with the recent report of different evolutionary mechanisms between lymph node metastasis *via* the lymph system and distance metastasis *via* the blood circulating system (Reiter et al., 2020).

### Cell Proliferation and Stem Cell–Related Drug-Resistance Information Detected in CTCs

**FIGURE 4:**
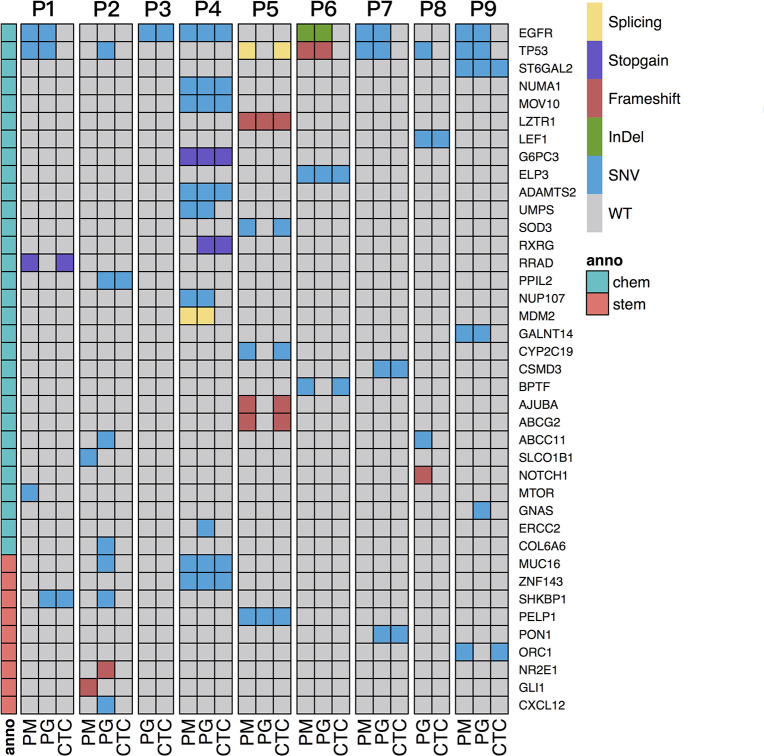
Screening of SNVs/Indels related to chemotherapy and stem cell features shared with PM and/or PG and CTC specimens of all the patients. The gray region represents wild-type. The blank denotes no sequencing reads covered. Sample types are shown on the bottom, and patient IDs are shown on the top of the graph. Mutation genes are listed on the right. The dark blue region represents mutation genes. Chemo denotes chemotherapy-related genes, and stem denotes stem cell feature–related genes, anno denotes annotation, shown on the left column.

To interpret drug-resistance genetic information in the CTCs of each patient, the SNVs/Indels of CTCs and progressive and primary specimens were blasted against a cancer chemotherapy database of PharmGKB annotations (see text footnote 1) and a gene list involved in 10 canonical oncogenic signaling pathways and 10 DNA damage repair pathways that were reported to have essential roles in tumor metastasis and drug resistance based on TCGA PanCancer Atlas Project (Knijnenburg et al., 2018; Sanchez-Vega et al., 2018) as described in section “Materials and Methods.” The SNVs/Indels were also screened by the keyword “stem cell” in the annotation results and then manually retrieved through public literatures to evaluate their relationship with cancer stem cell features. The results show that the two most frequent mutation genes were *EGFR* and *TP53* (Figure 4), which are both cancer-driver and drug-resistance genes in LC. Several cell cycle- and stem cell-related gene mutations were also identified to share between CTCs and primary or progressive specimens depending on different patients (Table 2).

**TABLE 2:** Genetic information identified in CTCs of platinum-drug resistance patients.

**Patient ID**	**Gene name** ^§^	**Sample (PM/PG)**	**Sample (CTCs)**	**Functions and pathways**	**Drug resistance**	**References**
P1	*SHKBP1*	PG	+	RTK/RAS and TGFβ-related signaling in stem cells	Erlotinib	Wang et al., 2019
	*RRAD*	PM	+	Ras-related GTPase signaling		Shang et al., 2016
P2	*SHKBP1*	PG	−	RTK/RAS and TGFβ-related signaling in stem cells	Erlotinib	Wang et al., 2019
	*PPIL2*	PG	+	Epithelial-mesenchymal transition (EMT)		Jia et al., 2018
P3	*EGFR*	PG	+	RTK/RAS signaling	Multi-drug resistance	
P4	*NUMA1*	PM, PG	+	Cell cycle	Multi-drug resistance	Qin et al., 2017
	*ZNF143*	PM, PG	+	Cell cycle, apoptosis	Multi-drug resistance	Izumi et al., 2010
	*MUC16*	PM, PG	+	Apoptosis	Cisplatin, gemcitabine	Lakshmanan et al., 2017; Kanwal et al., 2018
P5	*LZTR1*	PM, PG	+	Wnt signaling		Motta et al., 2019
	*PELP1*	PM, PG	+	Estrogen receptor signaling		Słowikowski et al., 2015; Wang and Bao, 2020
P6	*BPTF*	PM	+	Wnt signaling and TGFβ signaling		Liu et al., 2011
	*ELP3*	PM, PG	+	Chromatin organization, PI3K/ATK signaling	Multi-drug resistance	Xu et al., 2018b
P7	*CSMD3*	PG	+	Regulation of dendrite development	Etoposide	Qiu et al., 2019
	*PON1*	PG	+	Regulating stem-cell related *Nanog* expression		✩
P8	*TP53*	PG	+	Cell cycle arrest, apoptosis	Multi-drug resistance	
P9	*ORC1*	PM, PG	+	Cell cycle, stem cell related		Zhang et al., 2020

*PM, primary samples; PG, progressive samples; CTC, circulating tumor cells.*

*+, positive in CTCs; −, negative in CTCs.*

*^§^The genes’ full names were in the main text.*

*✩https://www.genecards.org/cgi-bin/carddisp.pl?gene=PON1.*

For P1, more somatic mutations were detected in the primary tumor (Figure 3B), and TMB decreased obviously in the progressive specimens (Figure 2B). An *EGFR* activity-regulating gene, *SHKBP1* (SH3KBP1 binding protein 1), mutation was identified in both progressive lymph nodes and CTCs. Its upregulation concomitantly induced activation of AKT through the TGFβ pathway and regulation of *EGFR* activity and was highly correlated with epithelial-mesenchymal transition (EMT) and resistance to erlotinib in osteosarcoma cancer stem cell–like cells (Wang et al., 2019). In addition, a nonsense mutation at C terminal (p.R263X) of *RRAD* gene was detected in both the primary tumor and CTCs. It is a tumor-suppressor gene and encodes a RAS-related glycolysis inhibitor and calcium channel regulator (*RRAD*), a small Ras-related GTPase that has been implicated in metabolic disease and several types of cancer. For example, its expression decreased in hepatocellular carcinoma (HCC) tumor tissues and played an important role in regulating aerobic glycolysis and cell invasion and metastasis of HCC (Shang et al., 2016). Therefore, both *SHKBP1* and *RRAD* probably contributed to tumor development and drug resistance through RTK/RAS-related regulations.

For P2, increased TMB and more chemotherapy drug–related gene mutations were detected in the progressive tumor (Figure 3C), including *TP53*, *CXCL12*, *MUC16*, *SHKBP1*, *ABCC11*, *CYP2C9*, and a nonsense mutation (p.Y158X) of *CYP26B1*, than those of the primary tumor, such as *SLCO1B1*. However, only mutations of *PPIL2* and *JAKMIP3* were identified in the progressive tumor and CTCs. Upregulation of *PPIL2* was reported to inhibit EMT and tumor invasion by interacting with the classical EMT transcription factor, *SNAI1*, to enhance its ubiquitin-dependent degradation in breast cancer (Jia et al., 2018). However, *JAKMIP3*’s role in LC is not known.

For P3 and P8, who only had progressive tumor and CTC samples (Figures 3D,I), the driver genes *EGFR* with p.L858R mutation and *LEF1* of the Wnt signaling pathway were detected, respectively, in both CTCs and progressive tumors of each patient. Mutation of *TAOK1* of the Hippo pathway and *KEAP1* of the NRF2 pathway in the progressive tumors of P3 as well as *NOTCH1* (frameshift deletion of p.R1824fs) of Notch pathway, *IRS1* and *RASAL2* of RTK-RAS pathway, *TP53* of p53/CPF pathway, and a multidrug-resistance gene *ABCC11* in the progressive tumors of P8 were also detected, indicating their roles are involved in the evolution of tumor progression and possibly also are involved in drug resistance.

Increased TMB, more progressive somatic mutations (Figure 3E), and more shared drug-resistance or cancer stem cell–related gene mutations were detected in P4 than in other patients (Figure 4 and Supplementary Table 3). For instance, *NUMA1* encodes nuclear mitotic apparatus protein 1, involved in mitotic prometaphase of the cell cycle. Its short isoform behaved as a putative tumor suppressor through regulating the expression of MYB proto-oncogene like 2 (*MYBL2*) and played an important role in the cell cycles and cancer relapse and drug resistance (Qin et al., 2017). *ZNF143* positively regulates tumor growth through transcriptional regulation of DNA replication and cell-cycle-associated genes (such as *CDC6*, *PLK1*, and *MCM*s) in multiple solid tumors, including in LCs (Izumi et al., 2010). *MUC16*, encodes mucin 16, a cell surface–associated protein, CA125, which is a biomarker in various cancers. The overexpression of *MUC16* induced by gene mutations was reported to affect LC cells, increasing their resistance to cisplatin and gemcitabine, promoting their growth, and enhancing their migration and invasion by downregulation of p53 (Lakshmanan et al., 2017; Kanwal et al., 2018). *MOV10*, encoding Mov10 RISC complex RNA helicase, was reported to be highly expressed with *POLR2A*, *MAPK3*, and *XAB2* in 95% of 54 lung adenocarcinoma (LUAD) cases with poor prognosis (Mao et al., 2020).

For P5, although there were hundreds of mutations identified in the CTCs and primary tumor specimens, only four mutations (VAF = 0.11—0.28) were shared ones (Figure 3F), probably due to sampling bias in the progressive specimen. Two important genes found in the CTCs and primary and progressive tumors: *LZTR1* (a frameshift deletion at p.T7fs) of the Wnt pathway and a transcription factor gene *PELP1* of the estrogen receptor (ER) signaling pathway, which both closely relate to cancer metastasis reported in LCs (Słowikowski et al., 2015; Motta et al., 2019; Wang and Bao, 2020). Two other mutations shared by the primary tumor and CTCs were cancer-driver gene *TP53* (splicing mutation at c.97-1G > T) of the p53/CPF pathway and *POLR2A* of NER (Supplementary Table 3).

For P6, many consistent mutations (76.9%) between primary and progressive hydrothorax specimens were observed (Figure 3G). Mutations of *BPTF*, encoding a bromodomain PHD-finger transcription factor, was identified in primary tumor and CTCs. It interacts with *SMAD2* and has functions of chromatin reorganization and transcriptional regulation through the Wnt signaling pathway downstream of the TGFβ pathway (Liu et al., 2011), indicating its potential role of tumor metastasis and relapse. The *ELP3* mutation was identified in both paired tumors and CTCs. It encodes the catalytic subunit 3 of the histone acetyltransferase elongator complex and contributes to transcript elongation and protein translation as well as chromatin organization and chromatin regulation/acetylation. Its overexpression promotes the migration and invasion of HCC and LC cells through the PI3K/AKT signaling pathway, suggesting elongator-driven metastasis in LC relapse and drug resistance (Xu et al., 2018b).

For P7, a dozen of the 53 somatic mutations shared between primary and progressive tumors (Figure 3H), such as *EGFR* (including hot spot mutation site p.L858R, and a non-canonical mutation p.D1014G), *TP53* and *SMAD4* mutations, indicating consistent tumor evolution in this patient. The mutations shared in CTCs and progressive tumors included *CSMD3* and *PON1*. *CSMD3* encodes CUB and Sushi multiple domains 3, a tumor suppressor. Its mutation is proved to resist to etoposide in SCLC (Qiu et al., 2019). *PON1* encodes paraoxonase 1, a member of the paraoxonase family of enzymes and exhibits lactonase and ester hydrolase activity. It is reported to increase *Nanog* expression by genome-RNAi experiment and contributes to cancer stem cell features.^3^

For P9, 137 somatic mutations were shared between primary and progressive tumors (Figure 3J), but only two mutations were shared with CTCs: DNA replication regulation gene *ORC1* and a transmembrane protein gene *ST6GAL2*. *ORC1* encodes origin recognition complex subunit 1, one of subunits of protein complex essential for the initiation of the DNA replication in eukaryotic cells. It is reported to interact with *CDC6* and *KAT7/HBO1* to regulate the cell cycle and be a cancer stem cell feature-related gene in LUAD (Zhang et al., 2020).

## Discussion

In addition to Pemetrexed, platinum-based chemotherapeutics are used for NSCLCs without targeted therapies. They mainly inhibit the division of cancer cells by causing DNA replication disorders and have a wide range of clinical applications in cancer therapies (Relling and Evans, 2015; Dilruba and Kalayda, 2016). However, the drug resistance effect is a main barrier to its treatment efficiency. CTCs are regarded as the seeds of tumor cells detached from primary tumors, and they migrate through the blood circulating system to distant locations and grow up to be metastatic tumors. The development of NGS technology help to provide large genetic information in CTCs isolated from drug-resistant patients for us to understand the drug-resistance mechanisms underlying tumor progression and explore new drug targets.

In this study, GO and KEGG enrichment analysis on the WES data showed SNVs/Indels harbored by CTCs had a distinct and relatively narrow functional profile focusing on the calcium/calmodulin binding membrane and signaling pathway as well as some essential genes of pathways in cancer in comparison with those of primary and progressive specimens (Supplementary Table 4). Furthermore, the SNVs/Indels shared among CTCs and primary and progressive specimens in the patients with progressive lung tumor tissue (33.3%) and hydrothorax specimens (4.4–15.6%; Figures 3D,E,G) were more than those with metastasis lymph nodes (0.6–11.8%; Figures 3B,C,F,H–J), agreeing that distant metastasis is carried by CTCs through fundamentally different evolutionary mechanisms from those of lymph node metastasis (Reiter et al., 2020).

Tumor cellular detoxification, proliferation, or apoptosis through coordination of DNA damage repair as well as drug hydration and dissociation and transmembrane and signaling transduction systems result in different responses to platinum drug treatment. Any gene mutations impairing these processes would definitely influence therapy efficiency. Genetic variations of different individuals are revealed to associate with platinum-based chemotherapy response and drug toxicity in different LC patients (Cui et al., 2017). Our studies also reveal complex and patient-specific somatic mutation features in the platinum drug–resistant advanced NSCLCs, particularly harbored by CTCs (Figure 4). The most frequent mutation genes were *EGFR* and *TP53*, which is previously reported in other drug-resistant cancers (Ye et al., 2016). In addition, many chemotherapy-drug transmembrane and metabolism genes, including *ABCC11*, *ABCG2*, *CYP26B1*, *CYP2C9*, *CYP2C19*, *ST6GAL2*, *RRAD*, etc., were identified in primary or progressive specimens, but only a few of them were detected in CTCs. Contrarily, the functions of shared mutations carried by CTCs were frequently enriched to cancer genes of regulation of RTK/RAS signaling; cell cycle and apoptosis; and TGFβ signaling pathways, particularly related to stem cell features, such as *SHKBP1* in P2, *PON1* in P7, and *ORC1* in P9 as well as *NUMA1*, *ZNF143* and *MUC16* of P4, *PELP1* of P5, and possibly *ELP3* and *BPTF* of P6 (Table 2), indicating the difference of intrinsic or acquired resistance between metastatic lymph nodes and CTCs in drug-resistant patients. The majority of these genes are derived from primary tumor samples (in 71.4% of patients except for two patients without the primary tumor samples) and a few genes (in 28.6% patients) might acquire mutations during tumor distance metastasis. The mutations or expressions of these genes are reported to have essential roles in cancer progression under platinum-based drug treatment and worthy to explore for new drug targets for antiplatinum drug resistance.

## Conclusion

Individual dissection of mutational profiles of CTCs and paired tumor samples of platinum-based patients based on NGS technology not only demonstrate a genetic spectrum through the blood circulating system distinct from that of the lymph circulating system, but also reveal recurrent essential mutation genes related to cell proliferation and stem cell features, which facilitates our understanding of the molecular mechanisms underlying platinum-drug resistance and cancer metastases through CTCs. Therefore, it is practical to use the CTC-NGS strategy for new drug target exploration if progressive tumor specimens are unavailable in clinical practice, particularly for advanced patients.

## Data Availability Statement

All the sequencing raw data used in this study have been uploaded to the Genome Sequence Archive depository database of National Genomics Data Center, China National Center for Bioinformation (https://ngdc.cncb.ac.cn/gsa) with the submission number HRA001083.

## Ethics Statement

The studies involving human participants were reviewed and approved by the Ethics Committee of Shanghai Chest Hospital (No. KS1740). The patients/participants provided their written informed consent to participate in this study. Written informed consent was obtained from the individual(s) for the publication of any potentially identifiable images or data included in this article.

## Author Contributions

RL designed the research and supervised the study. YW, XL, and HL performed the research. YC, BL, RW, and SW analyzed the data. BY, AG, WW, and AH managed the samples. YC, YW, SW, and RL wrote the manuscript. All authors contributed to the article and approved the submitted version.

## Conflict of Interest

YW, BL, XL, RW, HL, and SW are employees of Berry Oncology Corporation. The remaining authors declare that the research was conducted in the absence of any commercial or financial relationships that could be construed as a potential conflict of interest.

## Publisher’s Note

All claims expressed in this article are solely those of the authors and do not necessarily represent those of their affiliated organizations, or those of the publisher, the editors and the reviewers. Any product that may be evaluated in this article, or claim that may be made by its manufacturer, is not guaranteed or endorsed by the publisher.

## Footnotes

1
https://www.pharmgkb.org/downloads

2
http://www.bioconductor.org/

3
https://www.genecards.org/cgi-bin/carddisp.pl?gene=PON1
